# Association between frequency of breakfast intake before and during pregnancy and infant birth weight: the Tohoku Medical Megabank Project Birth and Three-Generation Cohort Study

**DOI:** 10.1186/s12884-023-05603-8

**Published:** 2023-04-19

**Authors:** Misato Aizawa, Keiko Murakami, Ippei Takahashi, Tomomi Onuma, Aoi Noda, Fumihiko Ueno, Fumiko Matsuzaki, Mami Ishikuro, Taku Obara, Hirotaka Hamada, Noriyuki Iwama, Masatoshi Saito, Junichi Sugawara, Nobuo Yaegashi, Shinichi Kuriyama

**Affiliations:** 1 Graduate School of Medicine, Tohoku University, Sendai, Japan; 2 Tohoku Medical Megabank Organization, Tohoku University, 2-1 Seiryo-Machi, Aoba-Ku, Sendai, 980-8573 Japan; 3 Department of Pharmaceutical Sciences, Tohoku University Hospital, Sendai, Japan; 4 Department of Obstetrics and Gynecology, Tohoku University Hospital, Sendai, Japan; 5 Suzuki Memorial Hospital, Iwanuma, Japan; 6 International Research Institute of Disaster Science, Tohoku University, Sendai, Japan

**Keywords:** Breakfast, Pregnant, Low birth weight, Japan

## Abstract

**Background:**

Low birth weight is associated with an increased risk of developing chronic diseases in adulthood, with a particularly high incidence in Japan among developed countries. Maternal undernutrition is a risk factor for low birth weight, but the association between the timing of food intake and infant birth weight has not been investigated. This study aimed to examine the association between breakfast intake frequency among Japanese pregnant women and infant birth weight.

**Methods:**

Of all pregnant women who participated in the Tohoku Medical Megabank Project Three Generation Cohort Study, 16,820 who answered the required questions were included in the analysis. The frequency of breakfast intake from pre- to early pregnancy and from early to mid-pregnancy was classified into four groups: every day and 5–6, 3–4, and 0–2 times/week. Multivariate linear regression models were constructed to examine the association between breakfast intake frequency among pregnant women and infant birth weight.

**Results:**

The percentage of pregnant women who consumed breakfast daily was 74% in the pre- to early pregnancy period and 79% in the early to mid-pregnancy period. The average infant birth weight was 3,071 g. Compared to women who had breakfast daily from pre- to early pregnancy, those who had breakfast 0–2 times/week had lower infant birth weight (β = -38.2, 95% confidence interval [CI]: -56.5, -20.0). Similarly, compared to women who had breakfast daily from early to mid-pregnancy, those who had breakfast 0–2 times/week had lower infant birth weight (β = -41.5, 95% CI: -63.3, -19.6).

**Conclusions:**

Less frequent breakfast intake before and mid-pregnancy was associated with lower infant birth weight.

**Supplementary Information:**

The online version contains supplementary material available at 10.1186/s12884-023-05603-8.

## Introduction

Low birth weight is associated with an increased risk of neonatal death [1], stunting in childhood [2], and chronic diseases in adulthood [3]. An analysis of birth weight data from United Nations member states from 2000 to 2015 showed that while the overall low birth weight rate decreased from 17.5% to 14.6%, the low birth weight rate in Japan increased from 8.6% to 9.5% [4]. Thus, the continued increase in low birth weight in Japan is an anomaly by global standards and requires intervention.

Factors influencing the decline in infant birth weight include low maternal weight [5], low gestational weight gain [6], hypertensive disorders of pregnancy [7], and the mother’s diet during pregnancy [8–11]. This suggests that maternal health and lifestyle affect the development of the fetus.

In addition, since night shifts during pregnancy may be associated with birth weight [12], pregnant women’s irregular circadian rhythm may affect infant birth weight [13]. Normalization of circadian rhythms requires morning sun exposure and breakfast [14, 15]. Disruption of circadian rhythms by skipping breakfast negatively affects obesity, metabolic functions [16, 17], memory [18], and depressive symptoms [19]. However, in Japan, the rate of skipping breakfast among 20–30-year-olds is as high as 25.8% [20], and the National Nutrition Survey has reported that skipping breakfast is a factor that increases the risk of unbalanced nutrient intake [21]. Pregnant women have been shown to skip breakfast at a high rate of 20–30%, suggesting an association with reduced nutrient intake [22] and an increased risk of gestational diabetes [23]. Furthermore, the fetus may be influenced by the circadian rhythm of maternal food intake [24]. Since not only “what and how much” the pregnant women eat but also “when” they eat affect infant growth, we hypothesized that less frequent maternal breakfast intake would decrease infants’ birth weight. However, to our knowledge, no study has examined the association between maternal breakfast intake frequency and infant birth weight.

This study aimed to investigate the association between the frequency of breakfast consumption among pregnant women in Japan and infant birth weight.

## Materials and methods

### Study design and population

**Fig. 1:**
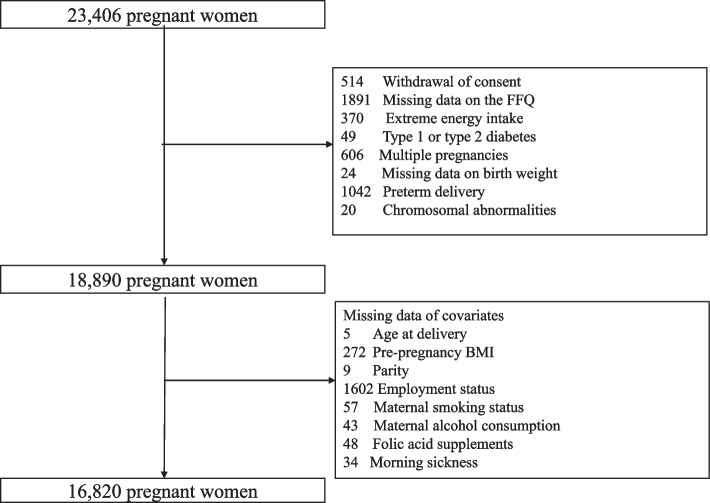
Study flow diagram of participants in the present analysis

The data were used from the Tohoku Medical Megabank Project Birth and Three-Generation Cohort Study (TMM BirThree Cohort Study), the details of which are described elsewhere [25, 26]. Pregnant women and their families were recruited from 2013 to 2017 from obstetrics and gynecology clinics or hospitals where deliveries had been scheduled. Approximately 50 obstetrics and gynecology clinics and hospitals in the Miyagi Prefecture participated in the recruitment process. A total of 23,406 pregnant women were enrolled, of whom 514 withdrew from participation, 1,896 had missing data on the Food Intake Frequency Questionnaire (FFQ), 369 had a total energy intake in the highest or lowest 1^st^ percentile (pre- to early pregnancy: < 605 or > 3828 kcal/day, early to mid-pregnancy: < 490 or > 3553 kcal/day), 49 had type 1 or 2 diabetes, 606 had multiple pregnancies, 24 had no medical record of birth weight, 1,042 had a preterm delivery, and 20 were excluded because of chromosomal abnormalities. Of the remaining 18,890 pregnant women, 2,070 were excluded because of missing covariates. Finally, the remaining 16,820 pregnant women were included in the analysis (Fig. 1). The TMM BirThree Cohort Study protocol was reviewed and approved by the Tohoku University Tohoku Medical Megabank Organization Ethics Committee (2013–1-103–1).

### Exposure variables

Frequencies of breakfast and other food intake were assessed using the 130-item self-administered semi quantitative FFQ. The FFQ used in this study had the response option “constitutionally unable to eat or drink” [27], which was found in the modified version of the FFQ used in the Japan Public Health Center-Based (JPHC) study [28, 29]. Data on the breakfast intake frequency among pregnant women were obtained from the first and second FFQs, which were administered in early pregnancy (0–13 weeks of gestation) and mid-pregnancy (14–27 weeks of gestation), respectively. The first FFQ assessed the frequency and quantity of foods and beverages consumed in the past 1 year, while the second FFQ assessed those consumed since the administration of the first FFQ. The breakfast intake frequency was assessed using the question, “How often do you eat breakfast?”. Categories of intake frequency in the response items were: less than once a month, 1–3 times a month, 1–2 times a week, 3–4 times a week, 5–6 times a week, and every day. The first three categories were combined 0–2 times a week because of the small number of participants. First, total energy and nutrient intake were calculated using data from the Standard Tables of Food Composition in Japan (Fifth Revised and Enlarged Edition 2005) [30]. Then, each food and nutrient consumed was energy-adjusted using the residual method [31].

### Outcome variables

Infant birth weight (g) was the outcome variable (continuous variable). Infant birth weight was obtained from birth records.

### Potential covariates

Based on previous studies, the following covariates were selected [5, 8–11, 23]: age at birth, body mass index (BMI) before pregnancy, smoking status, alcohol consumption, morning sickness, parity, employment status, insomnia, child sex, gestational week, folic acid supplements, and intake of energy, cereal, meat, seafood, bean, vegetables, and fruits. Age at delivery was categorized into four groups: < 25, 25–29, 30–34, and ≥ 35 years. Before pregnancy, BMI was classified into three groups: < 18.5, 18.5–24.9, and ≥ 25.0 kg/m^2^. The smoking status was categorized into four groups: never-smoked, quit before pregnancy, quit after pregnancy, and currently smoking. Alcohol consumption was categorized into three groups: never, former, and current. Morning sickness was categorized into four groups: never, nausea only, able to eat with vomiting, and unable to eat with vomiting. The employment status was categorized as employed or unemployed. Insomnia was defined as a score ≥ 6 on the Japanese version of the Athens Insomnia Scale [32]. Intake of total energy, cereal, meat, fish, beans, vegetables, and fruits were calculated using FFQ.

The variance inflation factor was <1.2, which was considered to be noncollinear [33].

### Statistical analysis

Statistical analyses were performed using the analysis of variance for continuous variables and the chi-square test for categorical variables to examine differences in characteristics of pregnant women according to their breakfast intake frequency. The association between maternal breakfast intake frequency and infant birth weight was evaluated with the multivariate linear regression analysis to calculate the regression coefficient β, 95% confidence interval (CI), and *p*-value for trend. Three models were constructed to examine these associations. Model 1 was a crude model. Model 2 was adjusted for age at birth, BMI before pregnancy, smoking status, alcohol consumption, morning sickness, parity, employment status, insomnia, child sex, and gestational week. Model 3 was adjusted for model 2 and folic acid supplements, intake of energy, grains, meat, fish, beans, vegetables, and fruits. To assess the goodness of fit of the model, coefficients of determination were calculated. Additional analyses were conducted, including risk factors for low birth weight (type 1 or type 2 diabetes, multiple pregnancies, preterm birth, and chromosomal abnormalities). Futhermore, because low energy intake is a risk factor for low birth weight [34], we performed a stratified analysis using energy intake quartiles. All statistical analyses were performed using SAS version 9.4 (SAS Institute Inc., Cary, NC, USA). Statistical significance was set at a *p*-value < 0.05.

## Results

Table 1 shows the characteristics of pregnant women and infants according to the breakfast intake frequency. The mean maternal age at birth was 31.3 ± 5.0 years. Frequencies of women who had breakfast daily were 74% and 79% in the pre- to early and early to mid-pregnancy periods, respectively. Compared to women who had breakfast every day, those who skipped breakfast more frequently had a lower age at delivery and higher rates of smoking, first childbirth, and insomnia. Women who skipped breakfast more frequently also had higher intakes of fat, vitamin A, niacin, vitamin B_12_, sugar, meat, confectionery, and alcoholic beverage (Supplementary Table 1). The mean infant birth weight was 3,071 ± 371 g.

**Table 1:** Characteristics of participants

	Frequency of breakfast intake (pre- to early pregnancy)										*p*–value^a^
	Total		Everyday		5–6 times/week		3–4 times/week		0–2 times/week		
	(*n* = 16,820)		(*n* = 12,442)		(*n* = 1673)		(*n* = 1165)		(*n* = 1540)		
**Pregnant women**	n (%) or mean (SD)										
Age at delivery (years)											
< 25	1494	(8.9)	757	(6.1)	223	(13.3)	197	(16.9)	317	(20.6)	< 0.001
25–29	4632	(27.5)	3160	(25.4)	550	(32.9)	398	(34.2)	524	(34.0)	
30–34	6174	(36.7)	4805	(38.6)	561	(33.5)	374	(32.1)	434	(28.2)	
≥ 35	4520	(26.9)	3720	(29.9)	339	(20.3)	196	(16.8)	265	(17.2)	
Pre-pregnancy BMI (kg/m^2^)											
< 18.5	2213	(13.2)	1604	(12.9)	220	(13.2)	168	(14.4)	221	(14.4)	< 0.001
18.5–24.9	12,427	(73.9)	9311	(74.8)	1230	(73.5)	814	(69.9)	1072	(69.6)	
≥ 25.0	2180	(13.0)	1527	(12.3)	223	(13.3)	183	(15.7)	247	(16.0)	
Smoking status											
Never	10,213	(60.7)	7987	(64.2)	942	(56.3)	603	(51.8)	681	(44.2)	< 0.001
Quit before pregnancy	3880	(23.1)	2938	(23.6)	385	(23.0)	248	(21.3)	309	(20.1)	
Quit after pregnancy	2333	(13.9)	1343	(10.8)	289	(17.3)	262	(22.5)	439	(28.5)	
Current	394	(2.3)	174	(1.4)	57	(3.4)	52	(4.5)	111	(7.2)	
Alcohol consumption											
Never	7737	(46.0)	5875	(47.2)	740	(44.2)	502	(43.1)	620	(40.3)	< 0.001
Former	5788	(34.4)	4156	(33.4)	614	(36.7)	415	(35.6)	603	(39.2)	
Current	3295	(19.6)	2411	(19.4)	319	(19.1)	248	(21.3)	317	(20.6)	
Parity ≥ 1	9016	(53.6)	7527	(60.5)	610	(36.5)	408	(35.0)	471	(30.6)	< 0.001
Employment status											
Employed	12,682	(75.4)	9275	(74.6)	1286	(76.9)	909	(78.0)	1212	(78.7)	< 0.001
Unemployed	4138	(24.6)	3167	(25.5)	387	(23.1)	256	(22.0)	328	(21.3)	
Morning sickness											
Never	2374	(14.1)	1698	(13.7)	249	(14.9)	173	(14.9)	254	(16.5)	< 0.001
Nausea only	7489	(44.5)	5713	(45.9)	684	(40.9)	492	(42.2)	600	(39.0)	
Vomiting, able to eat	5163	(30.7)	3769	(30.3)	553	(33.1)	363	(31.2)	478	(31.0)	
Vomiting, unable to eat	1794	(10.7)	1262	(10.1)	187	(11.2)	137	(11.8)	208	(13.5)	
Insomnia	5885	(35.0)	4105	(33.0)	636	(38.0)	476	(40.9)	668	(43.4)	< 0.001
Folic acid supplementation											
Yes	9460	(56.2)	6955	(55.9)	991	(59.2)	653	(56.1)	861	(55.9)	0.08
No	7360	(43.8)	5487	(44.1)	682	(40.8)	512	(44.0)	679	(44.1)	
Energy intake (kcal/day)	1635	(529)	1669	(517)	1600	(524)	1567	(570)	1451	(554)	< 0.001
**Infant**											
Birth weight (g)	3071	(371)	3076	(371)	3074	(357)	3044	(383)	3049	(371)	0.003
Boy	8606	(51.2)	6344	(51.0)	872	(52.1)	589	(50.6)	801	(52.0)	0.71

*BMI* Body mass index, *SD* Standard deviation

^a^Compared using the chi–square test for categorical variables and the analysis of variance for continuous variables

Table 2 shows the association between frequency of breakfast intake and infant birth weight among pregnant women. Compared with women who consumed breakfast daily pre- to early pregnancy, the regression coefficients (95% CI) for infant birth weight for women who consumed breakfast 5–6, 3–4 and 0–2 times a week were -2.1 (-21.0, 16.9), -32.1 (-54.4, -9.9) and -26.9 (-46.6, -7.3) respectively ( Model 1). After adjusting for covariates, the regression coefficients (95% CI) were -14.5 (-31.5, 2.5), -45.0 (-65.1, -25.0), and -44.3 (-62.3, -26.3), respectively (Model 2). Furthermore, after adjusting for dietary intake, the regression coefficients (95% CI) were -12.4 (-29.4, 4.6), -41.7 (-61.9, -21.6), and -38.2 (-56.5, -20.0), respectively (Model 3). Compared with women who consumed breakfast daily early to mid-pregnancy, the regression coefficients (95% CI) for infant birth weight for women who consumed breakfast 5–6, 3–4 and 0–2 times a week were -5.0 (-24.5, 14.5), -18.3 (-42.4, 5.8) and -36.5 (-60.2, -12.9) respectively ( Model 1). After adjusting for covariates the regression coefficients (95% CI) were -12.5 (-32.2, 2.7), -38.1 (-59.8, -16.4), and -49.0 (-70.6, -27.3), respectively (Model 2). Furthermore, after adjusting for dietary intake, the regression coefficients (95% CI) were -12.2 (-29.7, 5.4), -33.6 (-55.4, -11.8), and -41.5 (-63.3, -19.6), respectively (Model 3). Less frequent breakfast intake from pre- to early pregnancy and from early to mid-pregnancy was associated with a dose-dependent decrease in infant birth weight in all models (all P for trend =  < 0.001). The association between frequency of maternal breakfast intake and infant birth weight, including risk factors for low birth weight, was similar to that in the main analysis (Supplementary Table 2). The regression coefficients for variables other than frequency of breakfast intake have been included in Supplementary Table 3.

**Table 2:** Multivariate linear regression analysis for the association between breakfast intake frequency and infant birth weight

	Model 1		Model 2		Model 3	
	β	(95% CI)	β	(95% CI)	β	(95% CI)
**Frequency of breakfast intake**						
Pre- to early pregnancy^b^						
Everyday	Ref		Ref		Ref	
5–6 times/week	-2.1	(-21.0, 16.9)	-14.5	(-31.5, 2.5)	-12.4	(-29.4, 4.6)
3–4 times/week	-32.1	(-54.4, -9.9)	-45.0	(-65.1, -25.0)	-41.7	(-61.9, -21.6)
0–2 times/week	-26.9	(-46.6, -7.3)	-44.3	(-62.3, -26.3)	-38.2	(-56.5, -20.0)
P for trend^a^	< 0.001		< 0.001		< 0.001	
Early to mid- pregnancy^c^						
Everyday	Ref		Ref		Ref	
5–6 times/week	-5.0	(-24.5, 14.5)	-12.5	(-32.2, 2.7)	-12.2	(-29.7, 5.4)
3–4 times/week	-18.3	(-42.4, 5.8)	-38.1	(-59.8, -16.4)	-33.6	(-55.4, -11.8)
0–2 times/week	-36.5	(-60.2, -12.9)	-49.0	(-70.6, -27.3)	-41.5	(-63.3, -19.6)
P for trend^a^	< 0.001		< 0.001		< 0.001	

*CI* Confidence interval, *β* Regression coefficients

Model 1 is crude

Model 2 is adjusted for age at delivery, pre-pregnancy BMI, parity, work status, smoking, alcohol intake, morning sickness, insomnia, child sex and gestational week

Model 3 is adjusted for covariates and folic acid supplementation, intake of energy, cereal, meat, seafood, bean, vegetable, and fruit

^a^P for trend were calculated as trends across categories

^b^The coefficients of determination for Models 1, 2 and 3 were 0.0007, 0.21 and 0.21, respectively

^c^The coefficients of determination for Models 1, 2 and 3 were 0.0005, 0.21 and 0.21, respectively

Table 3 shows the results of a stratified analysis to determine whether the association between maternal breakfast intake and infant birth weight varied by energy intake. Compared with mothers who consumed breakfast daily prepregnancy to early pregnancy, the adjusted β (95% CI) of infant birth weight for mothers who consumed breakfast 0–2 times per week were -26.7 (-55.9, 2.4), -23.5 (-62.6, 15.7), -47.3 (-88.3, -6.4), and -64.9 (-108.8, -21.0) in the first, second, third, and fourth energy intake quartiles, respectively. Adjusted β (95% CI) for infant birth weight for mothers who consumed breakfast 0–2 times per week from early pregnancy to midpregnancy were -23.5 (-57.3, 10.3), -25.6 (-72.5, 21.3), -51.8 (-101.9, -1.6), and -66.1 (-122.9, -9.2) in the first, second, third, and fourth energy intake quartiles, respectively.

**Table 3:** Multivariate linear regression for the association between breakfast intake frequency during pregnancy and infant birth weight, stratified by energy intake

	Energy intake							
	First quartile		Second quartile		Third quartile		Fourth quartile	
**Frequency of breakfast intake**	**β**^**a**^	**(95% CI)**	**β**^**a**^	**(95% CI)**	**β**^**a**^	**(95% CI)**	**β**^**a**^	**(95% CI)**
Pre- to early pregnancy								
Everyday	Ref		Ref		Ref		Ref	
5–6 times/week	-6.2	(-39.1, 26.8)	3.9	(-29.1, 37.0)	-16.0	(-50.3, 18.3)	-26.3	(-62.7, 10.1)
3–4 times/week	-34.7	(-70.6, 1.2)	-48.4	(-88.4, -8.4)	-23.5	(-67.3, 20.3)	-58.1	(-101.8, -14.4)
0–2 times/week	-26.7	(-55.9, 2.4)	-23.5	(-62.6, 15.7)	-47.3	(-88.3, -6.4)	-64.9	(-108.8, -21.0)
Early to mid- pregnancy								
Everyday	Ref		Ref		Ref		Ref	
5–6 times/week	-10.4	(-43.4, 22.5)	-0.5	(–35.2, 34.3)	15.9	(-21.1, 53.0)	-40.6	(-76.9, -4.3)
3–4 times/week	-9.0	(-46.1, 28.2)	-54.3	(-96.5, -12.2)	-33.2	(-84.5, 18.1)	-30.8	(-80.0, 18.4)
0–2 times/week	-23.5	(-57.3, 10.3)	-25.6	(-72.5, 21.3)	-51.8	(-101.9, -1.6)	-66.1	(-122.9, -9.2)

*CI* Confidence interval, *β* Regression coefficients

^a^Adjusted for age at delivery, pre-pregnancy body mass index, parity, work status, smoking, alcohol intake, morning sickness, insomnia, folic acid supplementation, intake of cereal, meat, seafood, bean, vegetable, fruit, child sex, and gestational age

## Discussion

The present study showed that less frequent breakfast intake from pre- to mid-pregnancy was associated with lower infant birth weight. To our knowledge, this is the first study to examine the association between breakfast intake frequency among Japanese pregnant women and infant birth weight.

A few previous studies investigated breakfast intake among pregnant women. Pregnant Japanese women who skip breakfast consume significantly less protein, meat, and dairy products than those who eat breakfast daily [22]. These findings are consistent with the results of this study. Another Japanese study showed that pregnant women who skipped breakfast had a higher risk of developing gestational diabetes, suggesting that skipping breakfast may negatively affect glucose metabolism [23]. In a study on breakfast intake in adults, those who consumed breakfast daily had a lower risk of developing obesity, metabolic syndrome, hypertension, type 2 diabetes, and stroke compared to those who consumed breakfast less frequently [35, 36], and this association did not change after adjusting for dietary quality scores [35]. The present study showed that a low breakfast intake frequency among pregnant women might affect their health for themselves and for two generations, which is a novel finding.

Three mechanisms are plausible, although we did not examine the mechanism by which the breakfast intake frequency among pregnant women affected infant birth weight. The first possible mechanism is that the circadian rhythm is disrupted when a pregnant woman skips breakfast, which affects the growth of the fetus. Regulation of correct circadian rhythms requires light stimulation from morning sun exposure and regular food intake [14, 37]. Skipping breakfast can disrupt circadian rhythms by affecting the expression of clock and clock-regulated genes [38]. In a study involving adults, the body clocks of the eliminating organs remained delayed when breakfast was skipped, even after waking up in the morning [15]. In a study involving students who were not habitual breakfast-eaters, the 24-h rhythms of the heartbeat and sympathetic nervous system advanced by 1–3 h, and triglyceride and low-density lipoprotein cholesterol levels decreased when breakfast was consumed for 2 weeks [39]. In addition, the fetus begins to form its internal clock at approximately 20 weeks of gestation, using the mother’s circadian rhythm as a model [24, 40]. Sleep-deprived women have a higher risk of preterm delivery [41], and women who work rotating shifts have increased risks of spontaneous abortion, preterm delivery, and low birth weight [42, 43]. The circadian rhythm of pregnant women who skip breakfast may affect that of the fetus, which in turn may affect the birth weight of the fetus.

The second possible mechanism is the effect of infrequent breakfast intake on reproductive function. Skipping breakfast in female college students was associated with dysmenorrhea because of reduced uterine blood flow [44–46]. In a study using mice, deletion in utero of the clock gene *Bmal1*, which regulates the circadian clock, caused perinatal abnormalities, such as placental abruption [47]. Women who skip breakfast may have disrupted reproductive rhythms and ovarian and uterine dysfunctions [48]. Thus, infrequent breakfast intake by pregnant women may be associated with poor blood flow to the uterus and placenta and decreased infant birth weight.

The third possible mechanism is that infrequent breakfast intake increases inflammatory biomarkers and reduces child growth. In a study involving Chinese adults, habitually skipping breakfast was associated with increased C-reactive protein levels [49]. Maternal chronic inflammation is a possible cause of low birth weight [50]. Therefore, infrequent breakfast intake may increase inflammation and decrease infant birth weight.

A previous study showed that skipping breakfast was associated with unhealthy eating habits [51]. The present study showed that mothers who skipped breakfast had lower energy and nutrient intakes. The association between lower frequency of breakfast consumption and lower infant birth weight was found in the second, third, and fourth quartiles of energy intake, both in the prepregnancy to early pregnancy and early pregnancy to midpregnancy periods. These findings suggest that skipping breakfast and eating more at lunch and dinner before and during midpregnancy may have an effect on reduced infant birth weight, independent of energy and nutrient intakes.

## Implications

The results of this study suggest that increasing breakfast frequency from preconception to mid-pregnancy may increase infant birth weight. In Japan, the Dietary Guidelines for Pregnant Women Starting Before Pregnancy have been revised, highlighting the importance of improving dietary habits even before pregnancy [52]. However, despite the problem of the high rate of breakfast deprivation among young Japanese women, the dietary guidelines do not include any guidelines for breakfast intake. Therefore, for forming healthy eating habits, guidelines for eating behavior should be laid down [53], particularly for breakfast intake for pregnant women. Considering the results of this study, interventions focused on increasing the breakfast intake frequency before and in early pregnancy may be effective in preventing reduced infant birth weight.

## Limitations

This study has several limitations. First, this study was conducted in a small part of Japan, and the results cannot be generalized to other populations. However, the average age of participants in a national survey of pregnant women in Japan was 31.0 years [54], and that of participants in this study was almost the same at 31.3 years. Second, because the data on skipping breakfast were obtained from participants’ self-reported questionnaires, misunderstandings regarding skipping breakfast would have arisen. However, the percentage of Japanese pregnant women who do not eat breakfast ranges from 20 to 30% [22, 23] and is similar to the percentage in this study (pre- to early pregnancy, 25.9%; early to mid-pregnancy, 21.0%). Third, the type and amount of food consumed for breakfast are unknown. To better examine the association between skipping breakfast and infant birth weight, the frequency of breakfast intake and the type, amount, and time of day of the meal should be examined. Fourth, skipping breakfast is influenced by various social backgrounds, such as night shifts and time availability. The covariates adjusted for this study may not fully eliminate the effects of these backgrounds. However, the proportion of pregnant women in Japan who work night shifts is as small as 7.7% [23]. Fifth, we did not measure circadian rhythms, such as waking and sleeping times.

## Conclusion

Compared to daily breakfast intake, consuming breakfast less than 5 times a week from prepregnancy to midpregnancy was associated with lower infant birth weight. The pregnancy period is a good opportunity for mothers to review their nutritional status and change undesirable eating habits. Our finding suggests that breakfast intake before and during pregnancy may be important for the prevention of low birth weight.

## Supplementary Information

**Additional file 1:Supplementary Table 1.** Nutrient and food group consumption by breakfast intake frequency. **Supplementary Table 2.** Multivariate linear regression analysis for the association between frequency of breakfast intake during pregnancy and infant birth weight (*n*=18,307). **Supplementary Table 3.** Multivariate linear regression analysis of the association between breakfast intake frequency and infant birth weight.

## Data Availability

The TMM BirThree Cohort Study data that support the findings of this study are not publicly available due to them containing information that could compromise research participant consent. All inquiries about access to the data should be sent to the TMM (dist@megabank.tohoku.ac.jp).

## Abbreviations

BMI: Body mass index
FFQ: Food frequency questionnaire
TMM BirThree Cohort Study: Tohoku Medical Megabank Project Birth and Three-Generation Cohort Study
CI: Confidence interval
