# Supplementary material for: Association between frequency of breakfast intake before and during pregnancy and infant birth weight: the Tohoku Medical Megabank Project Birth and Three-Generation Cohort Study
Source: BMC Pregnancy Childbirth. 2023 Apr 19;23:268. doi: 10.1186/s12884-023-05603-8 (PMC10114420; doi:10.1186/s12884-023-05603-8)
Supplement: Supplementary file 1 — Additional file 1:Supplementary Table 1. Nutrient and food group consumption by breakfast intake frequency. Supplementary Table 2. Multivariate linear regression analysis for the association between frequency of breakfast intake during pregnancy and infant birth weight (n=18,307). Supplementary Table 3. Multivariate linear regression analysis of the association between breakfast intake frequency and infant birth weight. [file 12884_2023_5603_MOESM1_ESM.docx]

| **Supplementary Table 1.** Nutrient and food group consumption by breakfast intake frequency | | | | | | | | | | | | | |
| --- | --- | --- | --- | --- | --- | --- | --- | --- | --- | --- | --- | --- | --- |
|  | | Frequency of breakfast intake | | | | | | | | | | | |
|  | | Everyday | | | 5–6 times/week | | | | 3–4 times/week | | | 0–2 times/week | |
| **Nutrient and food group consumption^a^** | | **Mean** | | **(SD)** | **Mean** | | **(SD)** | | **Mean** | **(SD)** | | **Mean** | **(SD)** |
| Energy (kcal/d) | | 1669.3 | | (517.3) | 1600.3 | | (524.4) | | 1567.4 | (569.8) | | 1450.8 | (553.7) |
| Protein (g/d) | | 57.5 | | (9.8) | 55.6 | | (10.0) | | 55.6 | (10.4) | | 55.1 | (11.9) |
| Lipid (g/d) | | 57.0 | | (12.7) | 56.9 | | (13.1) | | 57.8 | (14.3) | | 57.6 | (14.7) |
| Carbohydrate (g/d) | | 213.8 | | (33.7) | 209.3 | | (35.6) | | 204.4 | (37.0) | | 202.5 | (37.5) |
| Dietary fiber (g/d) | | 10.3 | | (3.7) | 9.4 | | (3.2) | | 9.1 | (3.2) | | 8.8 | (3.1) |
| Sodium (mg/d) | | 3217.6 | | (936.2) | 3068.4 | | (925.5) | | 3095.5 | (961.4) | | 3030.8 | (960.2) |
| Potassium (mg/d) | | 2095.2 | | (515.0) | 1987.3 | | (495.7) | | 1992.2 | (498.4) | | 1963.7 | (511.7) |
| Calcium (mg/d) | | 491.2 | | (246.7) | 451.7 | | (232.4) | | 441.3 | (225.4) | | 435.5 | (234.4) |
| Magnesium (mg/d) | | 215.9 | | (43.4) | 205.0 | | (41.5) | | 203.5 | (40.6) | | 201.0 | (42.3) |
| Phosphorus (mg/d) | | 923.0 | | (190.0) | 881.9 | | (185.6) | | 878.6 | (190.2) | | 867.3 | (199.8) |
| Iron (mg/d) | | 6.6 | | (1.6) | 6.2 | | (1.4) | | 6.2 | (1.5) | | 6.1 | (1.5) |
| Zinc (mg/d) | | 7.0 | | (1.0) | 6.8 | | (1.1) | | 6.8 | (1.2) | | 6.7 | (1.3) |
| Copper (mg/d) | | 1.0 | | (0.2) | 0.9 | | (0.2) | | 0.9 | (0.2) | | 0.9 | (0.2) |
| Vitamin A (μgRAE) | | 505.7 | | (348.2) | 471.0 | | (311.8) | | 498.8 | (352.8) | | 504.9 | (376.2) |
| Vitamin D (μg/d) | | 5.4 | | (3.8) | 5.0 | | (3.3) | | 5.1 | (3.3) | | 5.0 | (3.4) |
| Vitamin K (μg/d) | | 227.4 | | (135.5) | 200.4 | | (105.1) | | 192.3 | (98.7) | | 186.9 | (94.4) |
| Vitamin E (mg/d) | | 5.8 | | (1.9) | 5.6 | | (1.8) | | 5.6 | (1.8) | | 5.5 | (1.8) |
| Vitamin B_1_ (mg/d) | | 0.8 | | (0.2) | 0.8 | | (0.2) | | 0.8 | (0.2) | | 0.8 | (0.2) |
| Vitamin B_2_ (mg/d) | | 1.1 | | (0.4) | 1.0 | | (0.3) | | 1.0 | (0.4) | | 1.0 | (0.4) |
| Vitamin B_6_ (mg/d) | | 6.5 | | (0.2) | 6.4 | | (0.2) | | 6.4 | (0.2) | | 6.4 | (0.2) |
| Vitamin B_12_ (μg/d) | | 4.4 | | (2.5) | 4.3 | | (2.6) | | 4.5 | (2.5) | | 4.6 | (2.5) |
| Folic acid (μg/d) | | 260.2 | | (91.0) | 241.5 | | (79.6) | | 240.0 | (81.0) | | 236.6 | (82.1) |
| Vitamin C (mg/d) | | 83.4 | | (43.2) | 78.3 | | (40.4) | | 79.0 | (41.9) | | 77.2 | (43.3) |
| Salt (g/d) | | 8.1 | | (2.4) | 7.8 | | (2.4) | | 7.8 | (2.4) | | 7.7 | (2.4) |
| Cereal (g/d) | | 433.3 | | (111.7) | 419.9 | | (113.0) | | 400.4 | (116.8) | | 388.3 | (120.3) |
| Potato (g/d) | | 24.8 | | (18.3) | 22.9 | | (18.5) | | 22.4 | (17.4) | | 22.0 | (16.9) |
| Sugar (g/d) | | 0.8 | | (2.2) | 1.1 | | (2.5) | | 1.3 | (3.0) | | 1.4 | (2.9) |
| Beans (g/d) | | 59.9 | | (60.4) | 57.2 | | (56.7) | | 52.8 | (59.8) | | 56.4 | (57.6) |
| Seed (g/d) | | 0.7 | | (2.9) | 0.7 | | (1.6) | | 0.8 | (2.0) | | 0.7 | (1.6) |
| Vegetables (g/d) | | 158.1 | | (107.4) | 141.5 | | (91.0) | | 140.6 | (85.9) | | 134.7 | (84.7) |
| Fruits (g/d) | | 146.9 | | (125.7) | 148.8 | | (132.4) | | 156.4 | (148.0) | | 156.5 | (163.0) |
| Mushroom (g/d) | | 10.7 | | (9.3) | 9.9 | | (8.4) | | 9.6 | (8.7) | | 9.0 | (7.8) |
| Algae (g/d) | | 6.5 | | (6.0) | 5.7 | | (5.5) | | 5.7 | (5.2) | | 5.3 | (4.8) |
| Seafood (g/d) | | 37.6 | | (29.7) | 35.8 | | (26.5) | | 36.8 | (28.8) | | 37.0 | (29.1) |
| Meat (g/d) | | 74.0 | | (38.9) | 79.4 | | (39.9) | | 85.0 | (42.9) | | 86.5 | (40.5) |
| Egg (g/d) | | 28.6 | | (26.6) | 27.0 | | (24.0) | | 27.5 | (30.6) | | 25.5 | (21.8) |
| Daily products (g/d) | | 202.9 | | (214.9) | 182.6 | | (195.4) | | 175.3 | (194.3) | | 169.9 | (185.7) |
| Fat and oil (g/d) | | 10.2 | | (4.6) | 9.9 | | (4.2) | | 10.0 | (4.0) | | 9.7 | (3.7) |
| Confectionery (g/d) | | 18.7 | | (18.3) | 19.9 | | (17.5) | | 20.0 | (16.5) | | 21.0 | (19.2) |
| Alcoholic beverage (g/d) | | 92.7 | | (224.5) | 128.7 | | (266.0) | | 153.3 | (307.9) | | 178.6 | (373.9) |
| SD: standard deviation  ^a^Energy-adjusted using the residual method. | | | | | | | | | |  | |  |  |
| **Supplementary Table 2.** Multivariate linear regression analysis for the association between frequency of breakfast intake during pregnancy and infant birth weight (n=18,307) | | | | | | | | | | |  |  |  |
|  | **Pre- to early pregnancy^b^** | | | | | **Early to mid-pregnancy^c^** | | | | |  |  |  |
|  | β^a^ | | (95% CI) | | | β^a^ | | (95% CI) | | |  |  |  |
| **Frequency of  breakfast intake** |  | |  | | |  | |  | | |  |  |  |
| Everyday | Ref. | |  | | | Ref. | |  | | |  |  |  |
| 5–6 times/week | -18.2 | | (-38.5, 2.1) | | | -18.9 | | (-39.2, 1.4) | | |  |  |  |
| 3–4 times/week | -28.1 | | (-53.1, -3.1) | | | -30.3 | | (-55.3, -5.4) | | |  |  |  |
| 0–2 times/week | -32.6 | | (-55.4, -9.8) | | | -34.8 | | (-57.5, -12.2) | | |  |  |  |
| P-for trend | <0.001 | |  | | | <0.001 | |  | | |  |  |  |
|  |  | |  | | |  | |  | | |  |  |  |
| Age at delivery |  | |  | | |  | |  | | |  |  |  |
| <25 years | Ref. | |  | | | Ref. | |  | | |  |  |  |
| 25–29 years | 5.3 | | (-19.8, 30.5) | | | 4.6 | | (-20.5, 29.8) | | |  |  |  |
| 30–34 years | 21.0 | | (-3.7, 45.8) | | | 19.7 | | (-5.0, 44.5) | | |  |  |  |
| ≥35years | 36.2 | | (10.6, 61.8) | | | 35 | | (9.4, 60.6) | | |  |  |  |
| Pre-pregnancy BMI (kg/m^2^) | | |  | | |  | |  | | |  |  |  |
| <18.5 | Ref. | |  | | | Ref. | |  | | |  |  |  |
| 18.5–24.9 | 89.3 | | (71.8, 106.8) | | | 89.4 | | (71.8, 106.9) | | |  |  |  |
| ≥25.0 | 158.6 | | (135.4, 181.9) | | | 159.6 | | (136.3, 182.9) | | |  |  |  |
| Smoking status |  | |  | | |  | |  | | |  |  |  |
| Never | Ref. | |  | | | Ref. | |  | | |  |  |  |
| Quit before pregnancy | 24.4 | | (10.0, 38.7) | | | 24.3 | | (9.9, 38.6) | | |  |  |  |
| Quit after pregnancy | 16.7 | | (-2.7, 36.1) | | | 16.9 | | (-2.5, 36.3) | | |  |  |  |
| Current | -65.1 | | (-111.1, -19.0) | | | -64.3 | | (-110.3, -18.3) | | |  |  |  |
| Alcohol consumption |  | |  | | |  | |  | | |  |  |  |
| Never | Ref. | |  | | | Ref. | |  | | |  |  |  |
| Former | -16.1 | | (-29.8, -2.4) | | | -16.1 | | (-29.7, -2.4) | | |  |  |  |
| Current | -15.9 | | (-31.9, 0.07) | | | -17.2 | | (-33.1, -1.3) | | |  |  |  |
| Parity |  | |  | | |  | |  | | |  |  |  |
| Once | Ref. | |  | | | Ref. | |  | | |  |  |  |
| More than twice | 42.3 | | (34.8, 45.8) | | | 42.9 | | (36.2, 47.0) | | |  |  |  |
| Employment status |  | |  | | |  | |  | | |  |  |  |
| Not employed | Ref. | |  | | | Ref. | |  | | |  |  |  |
| Employed | -33.5 | | (-47.1, -19.9) | | | -33.6 | | (-47.2, -20.0) | | |  |  |  |
| Morning sickness |  | |  | | |  | |  | | |  |  |  |
| Never | Ref. | |  | | | Ref. | |  | | |  |  |  |
| Nausea only | 15.7 | | (-2.3, 33.8) | | | 15.6 | | (-2.5, 33.6) | | |  |  |  |
| Vomiting, able to eat | 32.1 | | (12.9, 51.3) | | | 32.1 | | (12.8, 51.3) | | |  |  |  |
| Vomiting, unable to eat | 31.5 | | (7.0, 56.0) | | | 32.4 | | (7.9, 56.9) | | |  |  |  |
| Insomnia |  | |  | | |  | |  | | |  |  |  |
| Yes | Ref. | |  | | | Ref. | |  | | |  |  |  |
| No | 8.9 | | (3.6, 21.5) | | | 7.7 | | (4.9, 20.3) | | |  |  |  |
| Folic acid supplementation |  | |  | | |  | |  | | |  |  |  |
| Yes | Ref. | |  | | | Ref. | |  | | |  |  |  |
| No | -10.4 | | (-22.5, -1.6) | | | -10.4 | | (-22.4, -1.7) | | |  |  |  |
| Type 1 diabetes |  | |  | | |  | |  | | |  |  |  |
| Yes | Ref. | |  | | | Ref. | |  | | |  |  |  |
| No | 397.2 | | (218.5, 575.8) | | | 400.7 | | (222.0, 579.3) | | |  |  |  |
| Type 2 diabetes |  | |  | | |  | |  | | |  |  |  |
| Yes | Ref. | |  | | | Ref. | |  | | |  |  |  |
| No | -2.4 | | (-120.4, 115.6) | | | -1.0 | | (-119.0, 116.9) | | |  |  |  |
| Multiple pregnancies |  | |  | | |  | |  | | |  |  |  |
| Yes | Ref. | |  | | | Ref. | |  | | |  |  |  |
| No | 85.2 | | (70.4, 99.9) | | | 85.1 | | (70.4, 99.8) | | |  |  |  |
| Preterm delivery |  | |  | | |  | |  | | |  |  |  |
| Yes | Ref. | |  | | | Ref. | |  | | |  |  |  |
| No | 153.8 | | (121.6, 185.9) | | | 153.3 | | (121.2, 185.5) | | |  |  |  |
| Chromosomal abnormalities |  | |  | | |  | |  | | |  |  |  |
| Yes | Ref. | |  | | | Ref. | |  | | |  |  |  |
| No | 3.2 | | (-168.9, 175.4) | | | 5.6 | | (-166.6, 177.8) | | |  |  |  |
| Energy intake (kcal/day) | 0.02 | | (0.006, 0.03) | | | 0.02 | | (0.01, 0.03) | | |  |  |  |
| Cereal intake (g/day) | 0.1 | | (0.06, 0.17) | | | 0.1 | | (0.04, 0.2) | | |  |  |  |
| Meat intake (g/day) | 0.01 | | (-0.2, 0.2) | | | 0.05 | | (-0.1, 0.2) | | |  |  |  |
| Seafood intake (g/day) | 0.09 | | (-0.1, 0.3) | | | 0.08 | | (-0.2, 0.3) | | |  |  |  |
| Beans intake (g/day) | -0.04 | | (-0.1, 0.06) | | | -0.02 | | (-0.1, 0.08) | | |  |  |  |
| Vegetables intake (g/day) | -0.02 | | (-0.08, 0.04) | | | -0.006 | | (-0.08, 0.06) | | |  |  |  |
| Fruit intake (g/day) | 0.08 | | (0.03, 0.1) | | | 0.1 | | (0.05, 0.2) | | |  |  |  |
| **Child** |  | |  | | |  | |  | | |  |  |  |
| Gestational age (week) | 140.0 | | (136.2, 145.1) | | | 140.6 | | (136.2, 145.1) | | |  |  |  |
| Child sex |  | |  | | |  | |  | | |  |  |  |
| Girl | Ref. | |  | | | Ref. | |  | | |  |  |  |
| Boy | 104.5 | | (92.8, 116.2) | | | 104.4 | | (92.6, 116.1) | | |  |  |  |
| CI: confidence interval, β: regression coefficients, BMI: body mass index | | | | | | | | | | |  |  |  |
| ^a^Regression coefficients were adjusted for all covariates. | | | | | | | | | | |  |  |  |
| ^b^The coefficients of determination was 0.21. | | | | | | | | | | |  |  |  |
| ^c^The coefficients of determination was 0.21. | | | | | | | | | | |  |  |  |

| **Supplementary Table 3**. Multivariate linear regression analysis of the association between breakfast intake frequency and infant birth weight | | | | |
| --- | --- | --- | --- | --- |
|  | Pre- to early pregnancy^b^ | | Early to mid- pregnancy^c^ | |
|  | β^a^ | (95% CI) | β^a^ | (95% CI) |
| **Frequency of breakfast intake** |  |  |  |  |
| Pre- to early pregnancy |  |  |  |  |
| Everyday | Ref. |  | Ref. |  |
| 5–6 times/week | -12.4 | (-29.4, 4.6) | -12.2 | (-29.7, 5.4) |
| 3–4 times/week | -41.7 | (-61.9, -21.6) | -33.6 | (-55.4, -11.8) |
| 0–2 times/week | -38.2 | (-56.5, -20.0) | -41.5 | (-63.3, -19.6) |
| **Mother** |  |  |  |  |
| Age at delivery |  |  |  |  |
| <25 years | Ref. |  | Ref. |  |
| 25–29 years | 7.3 | (-12.1, 26.8) | 6.4 | (-13.1, 26.0) |
| 30–34 years | 20.8 | (1.6, 40.1) | 19.3 | (0.004, 38.6) |
| ≥35years | 33.0 | (12.8, 53.2) | 31.7 | (11.4, 51.9) |
| Pre-pregnancy BMI (kg/m^2^) |  |  |  |  |
| <18.5 | Ref. |  | Ref. |  |
| 18.5–24.9 | 97.1 | (82.1, 112.0) | 97.7 | (82.7, 112.6) |
| ≥25.0 | 187.1 | (167.5, 206.7) | 188.7 | (169.1, 208.3) |
| Smoking status |  |  |  |  |
| Never | Ref. |  | Ref. |  |
| Quit before pregnancy | 25.1 | (12.8, 37.5) | 24.6 | (12.2, 36.9) |
| Quit after pregnancy | 19.1 | (3.8, 34.5) | 17.8 | (2.4, 33.1) |
| Current | -71.1 | (-104.8, -37.5) | -71.5 | (-105.3, -37.8) |
| Alcohol consumption |  |  |  |  |
| Never | Ref. |  | Ref. |  |
| Former | -14.5 | (-26.1, -2.9) | -15.9 | (-27.5, -4.3) |
| Current | -12.3 | (-26.0, 1.4) | -14.9 | (-28.6, -1.3) |
| Parity |  |  |  |  |
| Once | Ref. |  | Ref. |  |
| More than twice | 41.8 | (34.4, 46.7) | 42.0 | (35.2, 46.9) |
| Employment status |  |  |  |  |
| Not employed | Ref. |  | Ref. |  |
| Employed | -36.5 | (-48.2, -24.8) | -37.2 | (-48.9, -25.5) |
| Morning sickness |  |  |  |  |
| Never | Ref. |  | Ref. |  |
| Nausea only | 10.6 | (-4.8, 25.9) | 11.2 | (-4.2, 26.5) |
| Vomiting, able to eat | 26.4 | (10.1, 42.6) | 27.0 | (10.7, 43.2) |
| Vomiting, unable to eat | 29.4 | (8.7, 50.1) | 30.5 | (9.8, 51.2) |
| Insomnia |  |  |  |  |
| Yes | Ref. |  | Ref. |  |
| No | 4.4 | (6.3, 15.1) | 4.2 | (6.5, 14.9) |
| Folic acid supplementation |  |  |  |  |
| Yes | Ref. |  | Ref. |  |
| No | 7.1 | (-3.1, 17.4) | 7.3 | (-2.9, 17.6) |
| Energy intake (kcal/day) | 0.01 | （0.005, 0.02) | 0.02 | (0.001, 0.03) |
| Cereal intake (g/day) | 0.1 | (0.005, 0.02) | 0.1 | (0.07, 0.2) |
| Meat intake (g/day) | 0.1 | (-0.06, 0.2) | 0.05 | (-0.09, 0.2) |
| Seafood intake (g/day) | 0.1 | (-0.1, 0.2) | 0.10 | (-0.09, 0.3) |
| Beans intake (g/day) | -0.02 | (-0.1, 0.07) | 0.01 | (-0.08, 0.1) |
| Vegetables intake (g/day) | -0.02 | (-0.07, 0.03) | -0.001 | (-0.06, 0.06) |
| Fruit intake (g/day) | 0.1 | (0.05, 0.1) | 0.09 | (0.04, 0.1) |
| **Child** |  |  |  |  |
| Gestational age (week) | 133.6 | (129.3, 138.0) | 134 | (129.2, 137.8) |
| Child sex |  |  |  |  |
| Girl | Ref. |  | Ref. |  |
| Boy | 110.6 | (100.6, 120.6) | 110.7 | (100.7, 120.7) |
| CI: confidence interval, β: regression coefficients, BMI: body mass index | | | | |
| ^a^Regression coefficients were adjusted for all other variables in the table. | | | | |
| ^b^The coefficients of determination was 0.21. | | | | |
| ^c^The coefficients of determination was 0.21. | | | | |
